# Grading and Reporting Health and Health Disparities

**Published:** 2009-12-15

**Authors:** Bridget C. Booske, Angela M. K. Rohan, David A. Kindig, Patrick L. Remington

**Affiliations:** University of Wisconsin School of Medicine and Public Health; Centers for Disease Control and Prevention/Council of State and Territorial Epidemiologists Applied Epidemiology Fellowship Program, Madison, Wisconsin. At the time of this research, Dr Rohan was affiliated with the University of Wisconsin School of Medicine and Public Health, Madison, Wisconsin; University of Wisconsin School of Medicine and Public Health, Madison, Wisconsin; University of Wisconsin School of Medicine and Public Health, Madison, Wisconsin

## Abstract

Report cards are widely used in health for drawing attention to performance indicators. We developed a state health report card with separate grades for health and health disparities to generate interest in and awareness of differences in health across different population subgroups and to identify opportunities to improve health. We established grading curves from data for all 50 states for 2 outcomes (mortality and unhealthy days) and 4 life stages (infants, children and young adults, working-age adults, and older adults). We assigned grades for health within each life stage by sex, race/ethnicity, socioeconomics, and geography. We also assigned a health disparity grade to each life stage. Report cards can simplify complex information for lay audiences and garner media and policy maker attention. However, their development requires methodologic and value choices that may limit their interpretation.

## Introduction

Report cards for reporting health care provider and plan performance began to proliferate in the 1990s and prompted a 1994 General Accounting Office study, which concluded that, although experts often disagree about their content, report cards can educate stakeholders (1). Public health has begun using report cards to draw attention to community health issues (2,3).

*Healthy People 2010* called for elimination of health disparities "among segments of the population, including differences that occur by gender, race or ethnicity, education or income, disability, geographic location, or sexual orientation" (4). To stimulate disparity reduction, the Institute of Medicine has called for increased awareness (5). Policy makers, however, have neither the time nor the inclination to digest large quantities of data (6), so reports that distill information and facilitate comparisons are often successful at drawing attention to issues. Interest in assessing Wisconsin's overall health and health disparity was stimulated by a group that assessed progress toward meeting two 2010 state health plan goals: promoting and protecting health for all and eliminating health disparities (7).

America's Health Rankings (8) has annually ranked states' health since 1990. Recently, these rankings have highlighted disparities without including them in their overall rankings. The states of Washington (9) and North Carolina (10) include health disparities in report cards but do not give overall grades for both health and health disparities.

Health disparities may occur across multiple population subgroups, but most health disparity measurement focuses on single domains, such as the Centers for Disease Control and Prevention's (CDC's) racial/ethnic disparity index (11). We developed an approach that assigns separate grades for both "health" and "health disparity"; we assessed disparities across multiple domains and published them in the Health of Wisconsin Report Card (12). In this article, we describe our methods and discuss issues in reporting and grading health and health disparities. These methods are applicable to other jurisdictions that seek to report on overall health and highlight inequalities in health.

## Assessing and Grading Overall Health

Two principles guided our selection of measures to compare Wisconsin’s overall health and the health of its subgroups to those of other states: 1) capturing both mortality and health-related quality of life (HRQOL) and 2) addressing different life stages. We collected age-adjusted mortality data from CDC WONDER (Wide-Ranging Online Data for Epidemiologic Research) (13) for the most recent years available at the time (2002-2004), by state, for each of 4 life stages: infants (aged <1 y), children and young adults (aged 1-24 y), working-age adults (aged 25-64 y), and older adults (aged ≥65 y).

We used unhealthy days as our HRQOL measure, an indicator of the extent of chronic and other diseases, for working-age and older adults. (Other HRQOL measures exist for children and young adults but not in state-level data.) We obtained the mean number of unhealthy days reported per month for adults aged 25 or older for the 3 most current years available at the time (2003-2005) from the Behavioral Risk Factor Surveillance System (BRFSS) (14). Unhealthy days are based on respondents' answers to 2 questions about their health in the past month: "How many days was your physical health poor?" and "How many days was your mental health poor?" We age-adjusted the measures for unhealthy days by using age groups 25 to 34, 35 to 44, 45 to 54, 55 to 64, 65 to 74, and 75 or older according to 2000 standard US population weights.

To develop overall grades for the state's health and health disparities, we assigned state-level grades for overall health, based on relative rather than absolute performance. We created grading scales (A to F) for each life stage on the basis of the distribution — mean and standard deviation — of state values for each measure. For each life stage and measure, we determined the mean and standard deviation. For each life stage, we then assigned a grade of C to each state for which the value of the measure fell within 0.5 SD of the national mean, a B or a D for states for which values were 0.5 to 1.4 SD below or above the mean, respectively, and an A or an F for states for which values were 1.5 SD or more below or above the mean, respectively (Table 1). We selected state distributions as our basis for grading because they represent observed (potentially achievable) values.

To calculate health grades for working-age and older adults, we averaged the grades for mortality and HRQOL. To determine a state's health grade, we averaged numerical equivalents for each of the 4 life-stage health grades (values of 4 for an A, 3 for a B, down to 0 for an F) to calculate an overall grade point average (GPA), with a range of 0 for worst health to 4 for best health. We then converted the overall GPA back to a grade: A, ≥3.75; A−, 3.50 to 3.74; B+, 3.26 to 3.49; B, 2.75 to 3.25; B− 2.50 to 2.74; C+, 2.26 to 2.49; C, 1.75 to 2.25; C−, 1.50 to 1.74; D+, 1.26 to 1.49; D, 0.75 to 1.25; and F, <0.75.

Because the life-stage grades for working-age and older adults were an average of the grades for mortality and HRQOL, and the overall health grade was an average of the 4 life-stage grades, 75% of the overall health grade was based on mortality (all ages) and 25% on HRQOL (working-age and older adults).

## Grading Health of Subpopulations

We used the same grading scales to assign grades for each population subgroup. We selected 4 population domains for which data were available to illustrate the different characteristics across which health disparities exist: sex, education, urbanization, and race/ethnicity.

We included educational attainment for adults aged 25 years or older as an illustration of socioeconomic disparities in health. We calculated mortality rates by education by using Public Use Mortality Files (15) and US Census population counts (16) for adults; for infants, we included maternal education (17). HRQOL (unhealthy days) by education was derived from BRFSS data.

Where someone lives can affect health, so we included an urbanization domain. Urbanization is a measure of the degree of urban character of the county in which a person lives. On the basis of the National Center for Health Statistics 6 urbanization classifications (18), we reported the "large central metro" classification as "large urban," combined "large fringe metro" and "medium metro" into "suburban/urban," combined "small metro" and "micropolitan" into "nonurban," and reported "noncore" as "rural."

For the race/ethnicity domain, where possible, we assessed health for 5 racial/ethnic groups, and all groups other than Hispanic/Latino were considered non-Hispanic ethnicity. We assigned racial and ethnic groups with a substantial population but no reliable data available a grade of I, for incomplete data.

## Grading Health Disparities

We developed a method for grading health disparities, building on an approach to assess disparity across multiple subgroups (within a single domain) by using an index of disparity (19). To calculate life-stage health disparity scores, we assigned numerical values to letter grades as described previously. We summed the differences between the values for the best subgroup grade and each subgroup and then divided this sum by the number of subgroups minus 1. We converted this new score to a percent scale by dividing it by 4 (the maximum scale value). The resulting disparity score ranged from zero percent disparity when all subgroups have the same grade to 100% disparity when 1 subgroup's grade is an A and all other subgroup grades are F's (Figure 1). In contrast to the health grades, where we assigned grades based on relative performance, we assigned the health disparity grades on the basis of an absolute scale.

**Figure 1:** Example of disparity scoring for infant mortality, Health of Wisconsin Report Card.

**Table:** 

		Mortality Rate	Subgroup Grade	Value of Grade	Difference From Best Grade (A = 4)
**Infants (aged <1 y)**					
Sex	Male	6.9	C	2	2
	Female	6.2	B	3	1
Education of mother	High school or less	9.0	D	1	3
	Some college/technical school	5.7	B	3	1
	College graduate	3.9	A	4	0
Type of county	Large urban	10.3	F	0	4
	Suburban/urban	4.8	A	4	0
	Nonurban	5.7	B	3	1
	Rural	7.0	C	2	2
Race/ethnicity	African American/black	17.6	F	0	4
	Asian	7.5	C	2	2
	Native American	9.0	D	1	3
	Non-Hispanic white	5.1	B	3	1
Total					24

**Table:** 


[Sum of differences from table above]	÷	(Total no. of subgroup grades minus 1)]	÷	Adjustment constant (transform to percentage)	=	Life-stage disparity score	Life-stage disparity grade
[24	÷	12]	÷	4	=	50%	D

**Table:** 

Disparity grading scale		
Disparity score	Grade	Interpretation
<15%	A	Very good
15%-30%	B	Good
30%-45%	C	Fair
45%-60%	D	Poor
>60%	F	Failing

We used the same approach for the 2 older life stages to calculate separate disparity scores for the 2 outcome measures (mortality and HRQOL). We then averaged these scores to derive a life-stage disparity score, which was then converted to an overall life-stage disparity grade (conversion in Figure 1). In the same way that we calculated an overall health grade for each state, we determined an overall health disparity grade for each state by averaging the 4 life-stage health disparity grades (with each life stage given equal weight) and then converting the average back to a grade by using the same conversion used for overall health.

Within each life stage, each population subgroup contributed equally to the calculation of the disparity grade. Since the 4 disparity domains had unequal numbers of subgroups, the domains were not equally weighted in the life-stage disparity grade. For example, the urbanization domain (with 4 subgroups) contributed twice as much to the disparity score as the sex domain (with 2 subgroups).

## Results

Although some states received grades of A for a particular life stage/outcome combination (for example, New Hampshire and Vermont received A's for infant mortality), no state received an A for overall health (Table 2). Furthermore, no state received an A for health disparity. New Hampshire received the best grades — a B+ for health and a B for health disparity — and Louisiana and West Virginia received the worst grades — F's for both health and health disparity.

**Figure 3:**
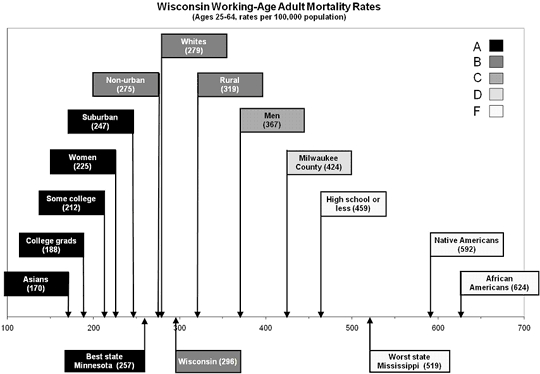
Example of graphic reporting of population subgroup grades. Reprinted from Booske BC, Kempf AM, Athens JK, Kindig DA, Remington PL. Health of Wisconsin report card. Madison (WI): University of Wisconsin Population Health Institute; 2007.

Since the reason for developing a report card was to communicate information to a lay audience, we used tabular and graphic formats (Figures 2 and 3) to display state-specific information to appeal to different people.

**Figure 2:** Example of tabular reporting of population subgroup grades. Reprinted from Booske BC, Kempf AM, Athens JK, Kindig DA, Remington PL. Health of Wisconsin report card. Madison (WI): University of Wisconsin Population Health Institute; 2007.

**Table:** 

		Percentage of Working-Age Adult Population	Unhealthy Days	Grades				
Working-Age Adults (25–64)			5.3		B			
Sex	Men	50%	4.5	A				
	Women	50%	6.2				D	
Education	High school or less	44%	6.2			C		
	Some college/technical school	31%	5.7			C		
	College graduate	25%	4.1	A				
Type of county	Urban	17%	6.1			C		
	Suburban/urban	32%	5.0		B			
	Nonurban	37%	5.2		B			
	Rural	14%	5.4		B			
Race/ethnicity	African American/black	5%	8.4					F
	Asian	2%	I					
	Hispanic/Latino	4%	8.0					F
	Native American	1%	I					
	Non-Hispanic White	88%	5.1		B			

## Discussion

America's Health Rankings has led the way in providing data on state health, but these rankings focus on overall health, not on disparities. Furthermore, most of its metrics are not life-stage specific. Because different factors operate within life stages and advocacy groups often focus on a particular life stage, the Health of Wisconsin Report Card was developed to examine relative state performance by life stage for both health and health disparity. Compared with direct data reporting, both ranking and report card grades result in information loss, but they reduce data to an understandable form for lay users and garner more attention.

Users of rankings often assume that the distance between successive items in a list is the same. For example, they may assume that the distance between the 48th and 49th states is the same as the distance between the 49th and the 50th, when in fact, the 48th and 49th states are similar but the 50th state is substantially worse. Grading cutoffs can be arbitrary, but an advantage of an appropriately designed grading scheme is that a D for the 49th state and an F for the 50th state show meaningful differences.

We found other advantages to using grades for measuring health disparity. For example, a reference group must be selected appropriately, but the recommended "best" subgroup approach (16) can be hindered by data reliability issues for subgroups with the best health outcomes. In addition, choosing whites as the reference group is not always well received by minority groups, and using a rate other than the "best" is complicated when rates in subgroups are better than the reference rate. Using the best grade as the reference avoided issues surrounding the reliability of individual point estimates.

A disadvantage of our approach is that an index ideally consists of a set of mutually exclusive and exhaustive population groups that are based on 1 or more characteristics of people in a population. A possible solution to this disadvantage could be to construct subpopulation groups on the basis of more than 1 characteristic. Another possible solution could be "to use individual data to measure the independent contributions of different characteristics, but that may even be more difficult with the current data, at least for mortality" (20). We acknowledge these concerns but recognize that insufficient sample size and data limitations preclude implementing these solutions.

We based several decisions, such as reporting both mortality and HRQOL as outcomes and valuing each equally (for the older life stages), on value choices. Further research could include sensitivity analyses to determine the effect of these choices. A group with different value systems could have come up with different cuts and assignments or balance and scores. Alternatively, others applying this same method might consider involving a wider audience in this value discussion.

Other value-based decisions included the cutoffs for assigning grades, equal weights for each life stage in calculating overall grades, use of an absolute scale for disparity grades, and equal weighting of all subgroups in disparity scoring, irrespective of number of subgroups or population size. This last item is controversial because, with this method, domains with more groups contribute disproportionately to the disparity score. We considered alternative approaches but decided in favor of this approach for ease of communication. In preparing material for nontechnical audiences, there is often a trade-off between increased precision and ease of communication.

Information on the relative size and rate for each subgroup was incorporated into the grade for overall health but not for disparity grades. Without information regarding which subgroup inequality might be of more concern, we weighted each group equally in calculating disparity grades. We reported population size for each subgroup to show that some subgroups that received failing grades (for example, those with less education) are much larger than others (for example, racial/ethnic minorities).

Lack of data for some subgroups changed the relative contribution to the overall disparity score, but by accounting for the number of subgroups with data available, the effect of missing data is minimized. Hispanic/Latino subgroup data were not available for mortality but were generally available for unhealthy days for working-age adults and sometimes for older adults (depending on subgroup size).

We hoped that media interest would stimulate policy makers to work with public health officials to examine health and health disparity data more carefully, to better understand the nature and extent of deficiencies in health and to begin to identify policy solutions. The publication of the report card did prompt substantial media coverage in the state (www.pophealth.wisc.edu/uwphi/pha/healthiestState/reportCard/2007.htm#media). Subsequent presentations at multiple venues appeared to stimulate discussion about health disparities and may initiate action to increase Wisconsin's grade above a D. Improving these grades is a candidate goal for the next state health plan, but we must also consider that some strategies to improve overall health may have little or no effect on disparities or even increase them (21). In conclusion, although report cards require making difficult methodologic and value choices, they are a useful tool for communicating complex information to lay audiences and for garnering media and policy attention.

## Figures and Tables

**Table 1:** Grading Scale and Distribution by Life Stage and Outcome Measure

Grade by Life Stage^a^	Age-Adjusted Mortality per 100,000 Population (2002-2004)^b^		Age-Adjusted HRQOL (2003-3005)^c^	

	Range	No. of States	Range	No. of States
**Infants (aged <1 y)**				
A	<5.0	3	—	—
B	5.0-6.3	13	—	—
C	6.4-7.7	18	—	—
D	7.8-9.1	13	—	—
F	>9.1	3	—	—
**Children and young adults (aged 1-24 y)**				
A	<31.9	3	—	—
B	31.9-42.3	15	—	—
C	42.4-52.7	17	—	—
D	52.8-63.1	11	—	—
F	>63.1	4	—	—
**Working-age adults (aged 25-64 y)**				
A	<259	1	<4.8	4
B	259-324	19	4.8-5.5	9
C	325-390	17	5.6-6.2	24
D	391-455	7	6.3-6.9	9
F	>455	6	>6.9	4
**Older adults (aged ≥65 y)**				
A	<4,278	1	<5.5	2
B	4,278-4,710	15	5.5-6.3	11
C	4,711-5,142	19	6.4-7.0	27
D	5,143-5,574	8	7.1-7.8	7
F	>5,574	7	>7.8	3

a A, ≥1.5 SD from the mean; B, 0.5 to 1.4 SD from the mean; C, −0.4 to 0.4 SD from the mean; D, −1.4 to −0.5 SD from the mean; F, ≤−1.5 SD from the mean.

b Mortality per 1,000 live births for infants. Data from CDC WONDER (Wide-Ranging Online Data for Epidemiologic Research).

c Health-related quality of life (HRQOL) was operationalized as mean number of unhealthy (physical and mental) days per month. HRQOL measures are unavailable for infants and unavailable for children and young adults at the state level. Data from the Behavioral Risk Factor Surveillance System.

**Table 2:** Overall State Grades for Health and Health Disparity

**Health Grade**	**Health Disparity Grade**	**No. of States**	**States**
B+	B	1	New Hampshire
B	B	2	Hawaii, Iowa
B	B−	3	Massachusetts, Minnesota, Vermont
B	C	4	Connecticut, New York, Rhode Island, Washington
B	C−	2	Colorado, Utah
B	F	3	Maryland, Michigan, Ohio
B−	C	3	California, Maine, North Dakota
B−	C−	1	New Jersey
B−	D	2	Nebraska, Wisconsin
C+	C−	1	Oregon
C+	D	1	South Dakota
C	C	1	Alaska
C	C−	2	Kansas, Montana
C	D	11	Arizona, Delaware, Florida, Idaho, Illinois, Nevada, New Mexico, Pennsylvania, Texas, Virginia, Wyoming
C−	F	3	Indiana, Missouri, North Carolina
D+	F	1	Georgia
D	D	1	Arkansas
D	F	4	Kentucky, Oklahoma, South Carolina, Tennessee
F	D	2	Alabama, Mississippi
F	F	2	Louisiana, West Virginia
